# Supplementary material for: Increases of Phosphorylated Tau (Ser202/Thr205) in the Olfactory Regions Are Associated with Impaired EEG and Olfactory Behavior in Traumatic Brain Injury Mice
Source: Biomedicines. 2022 Apr 7;10(4):865. doi: 10.3390/biomedicines10040865 (PMC9031269; doi:10.3390/biomedicines10040865)
Supplement: Supplementary file 1 [file biomedicines-10-00865-s001.zip › Supplemental information 복사본.pdf]

Supplemental Information

Figure S1. Activity levels are not significantly changed between control and TBI mice in Y-maze test. **a** Total distance and velocity measured during Y maze experiments. TBI mouse models exhibit a slight increase in physical activity compared to controls without reaching significance (distance, $p = 0.3058$; velocity, $p = 0.3913$). **b** Total frequency and duration in the starting arm measured during Y maze experiments. No significant difference was detected between controls vs. TBI mice (frequency, $p = 0.9595$; duration, $p = 0.4475$) [control, N=5; TBI, N=6].

Table S1. Table of animals and time line for experiments

Experiment	Mouse line (Gender)	Age of animal (at 1st TBI)	Number of animals	Timeline of experiment
Pathology	WT (male)	3 months	Control; N=5<br>TBI; N=5	3days after TBI
Y-maze	WT (male)	3 months	Control; N=5<br>TBI; N=6	3days after TBI
Buried food seeking test	WT (male)	3 months	Control; N=6<br>TBI; N=6	3days after TBI
Olfactory Oddball test	Thy1 (male)	3 months	Control; N=4<br>TBI; N=10	3-5days after TBI

Video S1. Searching activity for home bedding is altered between control and TBI mice in Y-maze test.
