# Supplementary figures and images for: Increases of Phosphorylated Tau (Ser202/Thr205) in the Olfactory Regions Are Associated with Impaired EEG and Olfactory Behavior in Traumatic Brain Injury Mice
Source: Biomedicines. 2022 Apr 7;10(4):865. doi: 10.3390/biomedicines10040865 (PMC9031269; doi:10.3390/biomedicines10040865)

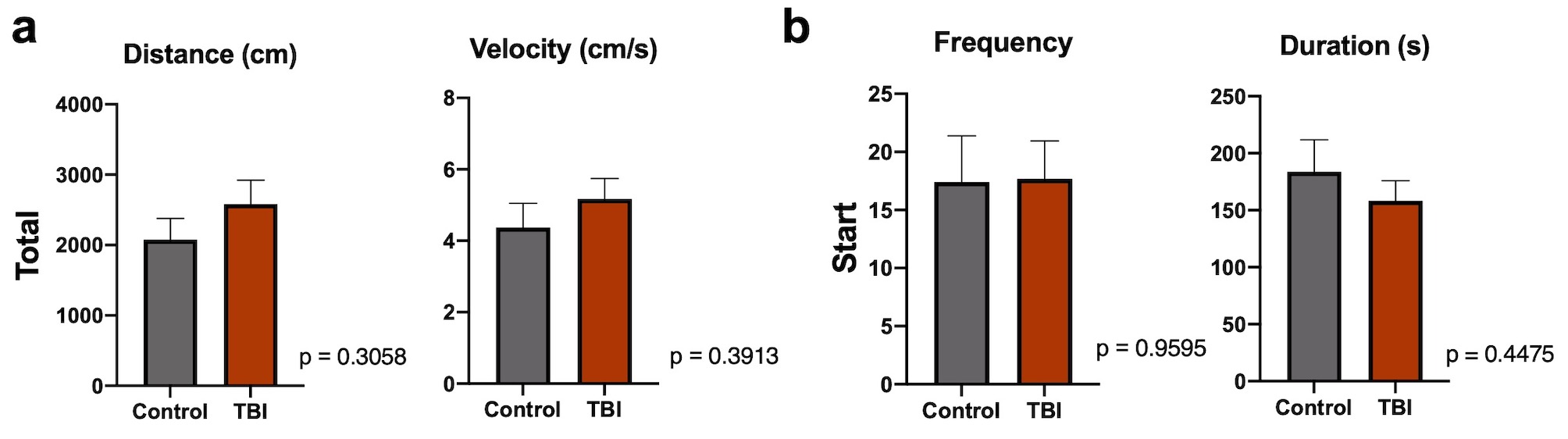

Supplement: Supplementary file 1 [file biomedicines-10-00865-s001.zip › Figure S1.jpg]
